# Serotonin and Dopamine Gene Variation and Theory of Mind Decoding Accuracy in Major Depression: A Preliminary Investigation

**DOI:** 10.1371/journal.pone.0150872

**Published:** 2016-03-14

**Authors:** Arielle Y. Zahavi, Mark A. Sabbagh, Dustin Washburn, Raegan Mazurka, R. Michael Bagby, John Strauss, James L. Kennedy, Arun Ravindran, Kate L. Harkness

**Affiliations:** 1 Department of Psychology, Queen’s University, Kingston, Ontario, Canada; 2 Department of Psychology, University of Toronto Scarborough, Toronto, Ontario, Canada; 3 Centre for Addiction and Mental Health, University of Toronto, Toronto, Ontario, Canada; INSERM / CNRS, FRANCE

## Abstract

Theory of mind–the ability to decode and reason about others’ mental states–is a universal human skill and forms the basis of social cognition. Theory of mind accuracy is impaired in clinical conditions evidencing social impairment, including major depressive disorder. The current study is a preliminary investigation of the association of polymorphisms of the serotonin transporter (SLC6A4), dopamine transporter (DAT1), dopamine receptor D4 (DRD4), and catechol-*O*-methyl transferase (COMT) genes with theory of mind decoding in a sample of adults with major depression. Ninety-six young adults (38 depressed, 58 non-depressed) completed the ‘Reading the Mind in the Eyes task’ and a non-mentalistic control task. Genetic associations were only found for the depressed group. Specifically, superior accuracy in decoding mental states of a positive valence was seen in those homozygous for the long allele of the serotonin transporter gene, 9-allele carriers of DAT1, and long-allele carriers of DRD4. In contrast, superior accuracy in decoding mental states of a negative valence was seen in short-allele carriers of the serotonin transporter gene and 10/10 homozygotes of DAT1. Results are discussed in terms of their implications for integrating social cognitive and neurobiological models of etiology in major depression.

## Introduction

The successful negotiation of our complex social world relies, at least in part, on having a “theory of mind”–an understanding of how others’ observable behaviors are connected with internal mental states, such as beliefs, emotions, and intentions [1]. Theory of mind (ToM) is a universal human skill with direct correlates in neural architecture [2,3], thereby implicating a biogenetic basis for its ontogeny and everyday use. Significant ToM deficits have been shown in clinical conditions associated with social dysfunction, including major depressive disorder [4], schizophrenia [5], and autism spectrum disorder [6]. That ToM is associated with these diverse clinical conditions suggests that variation in social cognition may represent a promising transdiagnostic endophenotype with biogenetic bases.

Connecting the broad social-cognitive task of ToM with biological mechanisms requires considering the specific processes that are involved in making mental state judgments [7]. Minimally, we can make a division between the cognitive processes that are associated with mental state *decoding* and mental state *reasoning* [3]. Mental state decoding refers to making judgments about mental states based upon immediately available information, such as facial expression or tone of voice (e.g., making a judgment that “she is confused” based on observing a furrowed brow in conversation). Mental state reasoning refers to the conceptual understandings regarding how mental states arise from experience and guide future action (e.g., reasoning that she is confused based on the knowledge that she is unfamiliar with the topic area). There is evidence that these two aspects of ToM rely on distinct neural systems. Specifically, mental state decoding relies on systems associated with the orbitofrontal cortex and the amygdala [8], whereas mental state reasoning relies on regions within the dorsal medial prefrontal cortex, and the temporal parietal junctures [9].

Several converging lines of evidence implicate dopamine and serotonin function in ToM abilities [10]. First, in terms of dopamine, the dorsal medial prefrontal cortex, which is consistently implicated in ToM reasoning, is a major target of mesocortical dopamine projections. Individual differences in dopamine function, as indexed by eyeblink rates, predict preschoolers’ performance on ToM reasoning tasks [11]. Further, studies with children and clinical groups have consistently reported that the Met allele of the catechol-*O*-methyl transferase (COMT) Val158Met SNP, which is associated with lower dopamine metabolism and thus higher synaptic dopamine levels and greater post-synaptic dopaminergic stimulation [12], predicts significantly superior executive function than the Val allele [13, 14]. Executive function is functionally linked to ToM reasoning [15].

Lackner et al. (2012) examined the association of the dopamine active transporter (DAT1), dopamine receptor D4 (DRD4), and COMT Val158Met SNP to ToM reasoning abilities in a sample of 73 preschool-aged children [11]. Specifically, children homozygous for the short (2- or 4-repeat) variants of the DRD4 VNTR, which are associated with enhanced prefrontal DRD4 expression [16], displayed superior performance to those with at least one long (6- or 7-repeat) variant. In contrast, COMT Val158Met and DAT1 VNTR were not significantly associated with ToM reasoning abilities. Further, a study examining several polymorphisms of the COMT gene to ToM reasoning in a sample of Han Chinese patients with schizophrenia found that the Val158Met polymorphism was not significantly associated with ToM reasoning performance, but two other functional polymorphisms were (rs2020917 and rs737865) [17]. To date, however, no work has examined genetic associations to theory of mind *decoding*. ToM decoding relies on a different cortico-limbic architecture from ToM reasoning, involving amygdala and orbitofrontal cortex. As such, it is possible that this skill is mediated by different neurotransmitter systems.

Second, in terms of serotonin, the serotonin-transporter-linked promoter region (5-HTTLPR) polymorphism of the serotonin transporter gene (SCL6A4) has been associated with social cognition more broadly construed. In healthy individuals, short (s)-allele carriers of the 5-HTTLPR, which are associated with reduced serotonin transporter (5-HTT) expression and 5-HT reuptake [18], show a hyperactivation of amygdaloid circuits in response to negative social stimuli (e.g., faces), and an attentional bias toward these stimuli relative to those homozygous for the long (l) allele [19, 20]. Further, studies in both human and non-human primates have found that s-allele carriers of the 5-HTTLPR have superior social-cognitive abilities than l/l homozygotes [21]. The 5-HTTLPR polymorphism has been associated with an amygdala-ACC-prefrontal cortical circuit that is involved in processing emotionally valenced stimuli [22]. As such, we suggest that this gene may be particularly relevant to individual differences in theory of mind *decoding*. However, to date, no studies have examined the relation of genes in the serotonin system to ToM abilities *per se*.

Major depressive disorder (MDD) may provide an especially sensitive population in which to examine the connection between dopaminergic and serotonergic functioning and ToM. Relative to healthy populations, individuals with MDD are significantly impaired in their ToM decoding [4] and reasoning [23] skills. At the same time, depressed individuals with a maternal history of MDD are significantly *more* accurate on tasks of ToM decoding than those without this history [24], and this inter-generational transmission of heightened social sensitivity may be genetically mediated [25]. Furthermore, MDD is associated with atypicalities in the neural circuits that are implicated in ToM decoding and that are heavily innervated by dopamine and serotonin [26]. For example, MDD patients, and those at risk by virtue of being s-allele carriers of the 5-HTTLPR, show lower amygdala and orbitofrontal cortical volumes than healthy individuals and those not at risk [27, 28]. Further, the effect of the s-allele of the 5-HTTLPR on amygdala responses to valenced social stimuli is stronger in MDD patients than controls [29]. Therefore, we suggest that the relation of polymorphisms in dopamine and serotonin genes to ToM accuracy may be stronger in individuals with MDD than in healthy controls.

The goal of the current study was to examine in a preliminary fashion the relation of polymorphisms in the SCL6A4, DAT1, DRD4, and COMT genes to ToM decoding in adults with a current diagnosis of MDD compared to adults with no psychiatric history. Numerous papers have been published on the issue of false positive findings and failures to replicate in candidate gene studies [30]. Our design included a number of theoretical, methodological, and statistical features that have been suggested to address the issues raised by small samples in targeted gene research [31]. First, we focus on a circumscribed skill (theory of mind decoding) that has been tied to a highly specific neuroanatomical substrate. As such, it is less heterogeneously determined than other broad phenotypes that have been examined in the targeted genetic and gene by environment literature. Second, our choice of genes is theory-driven based on imaging and preclinical data on the neuroanatomical substrates of theory of mind and social cognition. Third, we report bootstrapped confidence intervals over 1000 samples for all of our primary significant mean differences below to determine a greater measure of accuracy to our estimates [32]. The four genes listed above are the only ones that we examined in relation to theory mind in this sample, and were chosen based on our strong theoretical rationale.

We hypothesized that polymorphisms indicating greater dopamine and serotonin signaling would be significantly associated with superior ToM decoding accuracy. Further, we hypothesized that the s-allele of the 5-HTTLPR would be associated with a bias in the decoding of negatively valenced mental states. In contrast, given the role of DRD4 and DAT1 in DA signaling in mesolimbic reward pathways, we hypothesize that the long allele of DRD4 and the 9-allele of DAT1, both of which are associated with greater dopamine signaling, would be associated with a bias in the decoding of positively valenced mental states. Finally, we hypothesized that these relations would hold more strongly for the MDD group than for those with no history of MDD.

## Method

### Participants

This research was approved by the Health Sciences Research Ethics Board at Queen's University (#PSYC-058-06) and the Ethics Board at the Centre for Addiction and Mental Health (#65/2006). This investigation was conducted according to the principles expressed in the Declaration of Helsinki. Written informed consent was obtained from the participants. Participants ranged in age from 18–30 and were either full-time University students at the undergraduate or graduate level or were gainfully employed at the time of the study. None were inpatients and none had been diagnosed with a developmental delay. There is no evidence for diminished capacity in non-inpatient, young, ambulatory samples of individuals with major depressive disorder [33], and it is not in line with best practices to assume that a capacity assessment is necessary. Therefore, we judged that they all had the capacity to consent and we did not seek surrogate consent.

The current study included 96 young adults recruited as part of a larger study on gene-environment interaction in depression [34]. Participants were recruited from newspaper advertisements and clinician referrals in the Greater Toronto Area (*n* = 51; 39 women; 22 depressed) and Kingston, Ontario (*n* = 45; 30 women; 16 depressed). Those in Toronto were significantly older than those in Kingston (*M*s = 23.08, 20.36; *SD*s = 2.98, 2.32; *t*[94] = 4.95, *p* = .001). Participants at the two sites did not differ in terms of accuracy on the ToM task, or allelic distribution (all *p*s > .29). Further, results of all of the primary analyses below were unchanged when site was included in the models as a covariate. Therefore, data from the two sites were collapsed for analyses, and the uncontrolled analyses are presented below for parsimony and ease of interpretation.

Depressed participants met Diagnostic and Statistical Manual for Mental Disorders (DSM-IV-TR) [35] criteria for a current episode of MDD. Exclusion criteria were a current or past diagnosis of bipolar disorder, a psychotic disorder, or substance dependence. Participants in the non-depressed group had no current or past psychiatric diagnosis. All participants were fluent in reading English.

### Measures

#### Diagnostic and symptom status

Current and lifetime diagnoses were assessed with the Structured Clinical Interview for DSM-IV-TR Axis I Disorders (SCID-I/P) [36]. The SCID-I/P was administered by doctoral-level interviewers who were trained and supervised by the third (Toronto) or senior (Kingston) authors. To assess the severity of depression symptoms, participants completed the 21-item self-report Beck Depression Inventory-II (BDI-II) [37], and were administered the 17-item clinician-rated Hamilton Rating Scale for Depression (HRSD) [38] by doctoral student interviewers. The BDI-II and HRSD have documented good psychometric properties and are the most widely used measures of depression severity [39].

#### Theory of mind decoding

**Fig 1:**
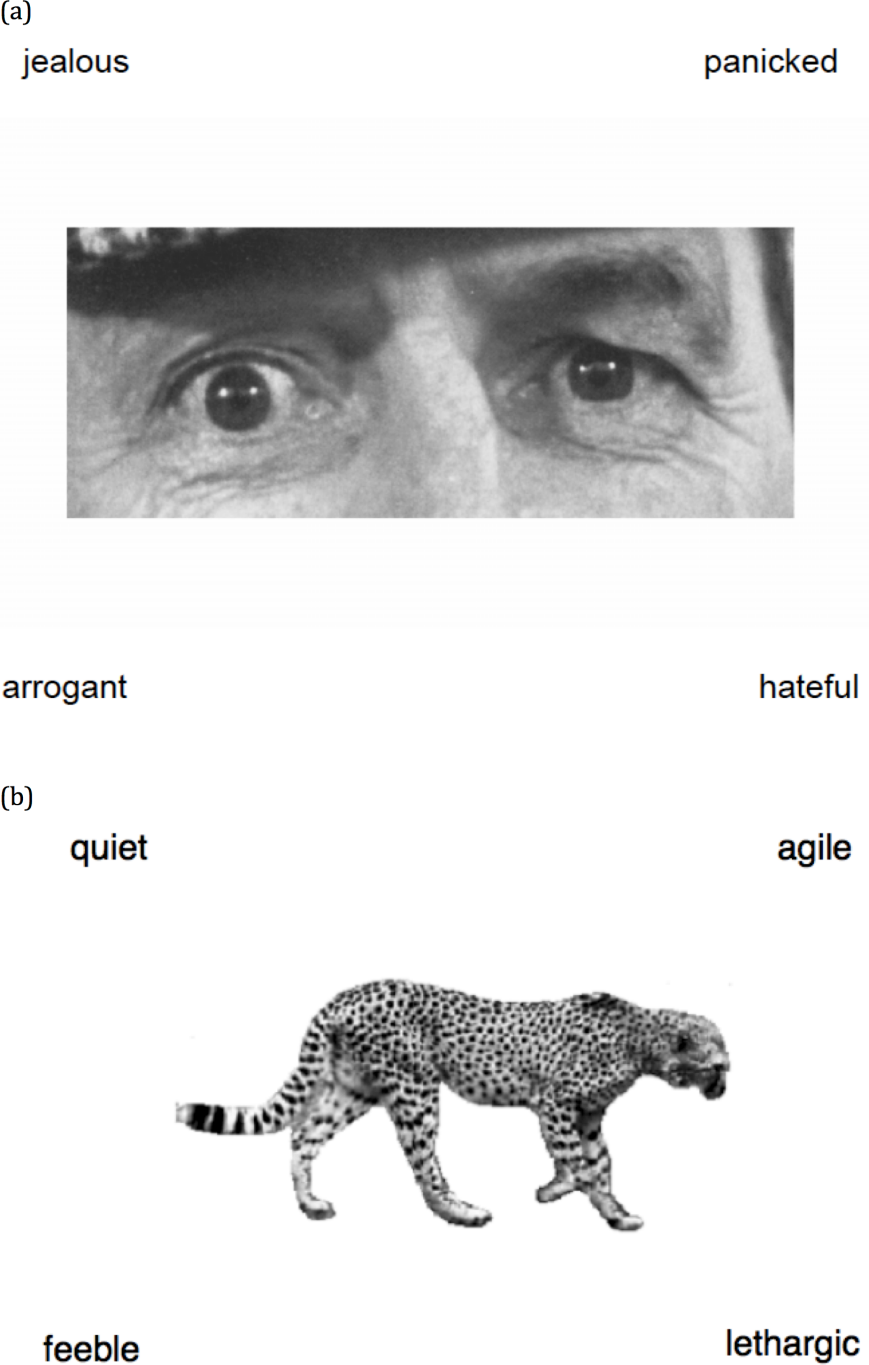
Sample Items from the (a) Eyes Task and (b) Animals Task.

The “Reading the mind in the eyes–revised version” task (Eyes task)[6] is the most widely used measure of ToM decoding in adults (Fig 1A). In the task, participants sequentially view 36 black-and-white magazine photographs (15 cm x 6 cm) of the eye region of a face from just above the eyebrows to halfway down the nose bridge. Participants select one of four mental-state adjectives (the standardized correct response and three distractors) that best represent the mental state of the person in the photograph by pressing one of four colored keys (S, X, K, M) that are spatially analogous to the adjectives’ location. The location of the standardized correct adjective is counter-balanced across the trials. The Eyes task stimuli have been classified into three valence categories: positive (e.g., “Friendly”), negative (e.g., “Upset”), and neutral (e.g., “Reflective”) [40]. Accuracy on the task is defined as the percentage of items on which the participant selected the target adjective. Response latencies are recorded in milliseconds (ms). This task is advantageous for examining individual differences because it is quite difficult and relies on sensitive decoding of the subtle social stimuli presented in eye expressions

#### Control Task

The Animals task [40] controls for differences between groups in task demands (e.g., choosing an item out of an array) and low-level perceptual processing (Fig 1B). Participants view 12 black-and-white photographs of animals that are surrounded by four adjectives, and select the adjective that best describes the animal in a format similar to the Eyes task.

### Procedure

At the Toronto site, participants completed all study measures and provided a blood sample during one assessment session. At the Kingston site, participants had provided a saliva sample at a previous session, and completed all of the study measures at a subsequent session. During the experimental task, participants first completed a practice trial followed by items of the Eyes and Animals tasks randomly combined in a single block of 48 trials (36 items for the Eyes and 12 items for the Animals). Participants were asked to respond as quickly and accurately as possible. Participants were provided with a monetary compensation of $10.00 per hour for their time.

#### Genotyping

Extraction and genotyping of the DNA was performed at the Neurogenetics Laboratory at the Centre for Addiction and Mental Health in Toronto. DNA from blood samples (25 mL preserved in EDTA) was extracted manually using a high salt method. Saliva samples (~2 mL) were collected from subjects in an Oragene OG-500 DNA kit (DNA Genotek, Ottawa, ON) and DNA was extracted as per manufacturer’s instructions. DNA was analyzed for the SLC6A4 LPR, DAT1VNTR, COMT Val158Met, and DRD4 VNTR variants according to standard procedures [41]. Genotyping of 10% of samples from each run were replicated for quality control purposes.

There was one assay failure for DAT1 and three for DRD4. Further, for DAT1, 3 participants had uncommon allelic variants (‘6’ and ‘11’). These participants were excluded from analyses with the relevant gene. Allelic distribution for the SCL6A4 5-HTTLPR was l/l (*n* = 28), s/l (*n* = 42), and s/s (*n* = 26), which did not differ significantly from Hardy-Weinberg equilibrium (χ^2^ = 1.49, *p* = .49). Consistent with previous research, we compared l/l homozygotes (*n* = 28) to those with at least one s allele (*n* = 68) [22]. Allelic distribution for the DAT1 gene was 10/10 (*n* = 57), 9/10 (*n* = 31), and 9/9 (*n* = 4), which did not differ significantly from Hardy-Weinberg equilibrium (χ^2^ = .73, *p* = .93). Given the small number of 9 homozygotes, we compared 10/l0 homozygotes (*n* = 57) to 9-variant carriers (*n* = 35). We classified those with DRD4 VNTR variants 2–5 as ‘s-short’ and 6–10 as ‘l-long’ [11]. DRD4 VNTR allelic distribution was s/s (*n* = 62), s/l (*n* = 29), l/l (*n* = 2), which did not differ significantly from Hardy-Weinberg equilibrium (χ^2^ = .43, *p* = .51). Given the small number of long homozygotes, we compared DRD4 s/s homozygotes (*n* = 62) to long carriers (*n* = 31). Finally, allelic distribution for the COMT Val158Met was Val/Val (*n* = 39), Val/Met (*n* = 38), and Met/Met (*n* = 19), which did not differ significantly from Hardy-Weinberg equilibrium (χ^2^ = 2.85, *p* = .09). Consistent with the role of the Met allele in promoting cognition, we compared Val/Val homozygotes (*n* = 39) to those with at least one Met allele (*n* = 57).

Previous studies have found very high concordance between whole blood and saliva methods of DNA collection [42, 43]. In the current sample we found no significant differences across the Toronto (blood) and Kingston (saliva) samples in terms of the allelic distribution of the 5-HTTLPR, χ^2^(2) = .16, *p* = .92, DAT1 VNTR, χ^2^(3) = .71, *p* = .87, DRD4 VNTR, χ^2^(2) = .26, *p* = .88, or COMT, χ^2^(2) = 2.18, *p* = .34. Further, as noted above, results of our primary analyses were robust when controlling for site.

### Data Analysis

Our analytic procedure involved four general linear mixed models. The within-subject factor in each model was Eyes task valence (positive, negative, neutral). The between-subject factors were dummy-coded depression group (0-non-depressed; 1-depressed) and dummy-coded 5-HTTLPR, DAT1, DRD4, and COMT genotypes, respectively. Significant interactions were followed up with pairwise comparisons based on estimated marginal means and adjusted for multiple tests using the Sidak correction.

## Results

### Demographic and Clinical Group Differences

The MDD and control groups did not differ significantly on sex, age, education, or ethnicity (all *p*s > .10; see Table 1). As expected, the MDD group scored significantly higher on the BDI-II and the HRSD than the non-depressed group. Six individuals in the MDD group were currently taking anti-depressant medication, which affects serotonergic and dopaminergic neurotransmission. Excluding these individuals did not change the primary analyses (analyses available by request).

**Table 1:** Sample Characteristics Stratified by Depression Group.

Variable	Non-Depressed (n = 58)	Depressed (n = 38)	*t* or χ^2^
Sex: Female n (%)	44 (76)	25 (66)	1.15
Age *M* (*SD*)	21.54 (2.87)	22.34 (3.17)	1.43
Years of Education *M* (*SD*)	15.38 (2.44)	14.97 (2.51)	.79
Ethnicity n (%)			7.88
White	35 (60)	25 (66)	
Asian	15 (26)	4 (10)	
African-Canadian	2 (3)	4 (10)	
First Nations	0	2 (5)	
Hispanic	0	1 (3)	
Other	6 (10)	2 (5)	
BDI *M (SD)*	3.09 (3.86)	29.92 (7.74)	19.60*
HRSD *M (SD)*	1.32 (2.39)	15.66 (6.58)	14.95*
Number of episodes *M* (*SD*)		1.55 (1.06)	
Age at first onset *M* (*SD*)		19.42 (4.21)	
Comorbidity: Yes n (%)		15 (40)	

* *p* < .001

No evidence was found for significant covariation of the 5-HTTLPR, DAT1, DRD4, or COMT genotypes (all *p*s > .20). Further, the depressed and non-depressed participants were not significantly differentially distributed across the 5-HTTLPR, DAT1, DRD4, or COMT genotypes (all *p*s > .07). However, more participants of non-White ethnicity were homozygous for the 10-allele of the DAT1 gene than heterozygous or homozygous for the 9-allele (53% vs. 14%; χ^2^[1] = 13.53, p < .001). Further, more women were s/s carriers of the DRD4 gene than l-allele carriers (82% vs. 52%; χ^2^[1] = 9.64, p = .002). Similarly, more women were Val/Val carriers of the COMT gene than Met-allele carriers (85% vs. 63%; χ^2^[1] = 5.27, p = .02). Finally, there was no significant relation of overall eyes task accuracy or accuracy on positive, negative, or neutral eyes with age, ethnicity, or BDI-II or HRSD scores (all *p*s > .05). However, higher education was significantly related to higher accuracy on the neutral-valenced eyes, *r*(94) = .26, *p* = .01. Results of the primary analyses were unchanged when controlling for education and, thus, the uncontrolled analyses are presented below for parsimony and ease of interpretation. However, sex and ethnicity were included as covariates in the analyses of the dopamine genes.

### Preliminary Analyses of Control Task Accuracy and Response Time

Accuracy and response times on the Eyes and Animals task by depression group are presented in Table 2. Eyes task accuracy was significantly correlated with Animals task accuracy, *r*(94) = .33, *p* = .001. Further, a series of four 2(depressed group) x 2(genotype for each of the four genes, respectively) ANCOVAs including Animals task accuracy as the dependent variable revealed main effects of genotype in two of these models. Specifically, those homozygous for the short allele of DRD4 were significantly less accurate than those heterozygous for the long allele, *M*s = 74.84, 82.84; *SD*s = 18.03, 13.26; *F*(1, 87) = 5.20, *p* = .02, η^2^ = .06. In addition, those homozygous for the Val allele of COMT were significantly less accurate than those heterozygous for the Met allele, *M*s = 71.41, 81.39; *SD*s = 19.15, 13.75; *F*(1, 90) = 4.60, *p* = .04, η^2^ = .05. However, no evidence for significant group or genotype by group effects emerged for any of the genes (all *p*s > .22). Results of the primary analyses below were unchanged when controlling for Animals task accuracy and, thus, the uncontrolled analyses are presented below for parsimony and ease of interpretation.

**Table 2:** Means and Standard Deviations for Eyes Task and Animals Task by Depression Group.

Variable	Non-Depressed (n = 58)	Depressed (n = 38)	*t*
Positive Eyes Task Accuracy	72.12 (15.72)	74.89 (17.49)	.81
Negative Eyes Task Accuracy	69.26 (16.67)	69.89 (17.50)	.18
Neutral Eyes Task Accuracy	71.96 (13.19)	66.24 (12.30)	2.13*
Positive Eyes Task RT	4229.71 (1882.19)	4676.79 (2004.05)	1.11
Negative Eyes Task RT	4189.64 (2083.97)	4685.86 (1787.08)	1.20
Neutral Eyes Task RT	4246.30 (1695.87)	4876.82 (2012.36)	1.65
Animals Task Accuracy	78.87 (16.88)	75.00 (16.63)	1.10
Animals Task RT	3437.12 (1469.70)	4645.37 (1974.65)	3.43**

RT: Response Time

* *p* < .05

** *p* < .01

Median response times (RT) on the positive, negative, and neutral Eyes task items were not associated with accuracy on the positive, *r*(94) < .005, *p* = .99, negative, *r*(94) = .04, *p* = .66, and neutral, *r*(94) = .03, *p* = .77, items, respectively, and the results of the primary analyses below were unchanged when controlling for response times on the respective valence categories. Further, we conducted a series four of 2(depressed group) x 2(genotype of each of the four genes, respectively) x 3(valence) mixed-design ANCOVAs including Eyes task response time as the dependent variable. Across all four models there was no evidence for significant genotype by RT valence (all *p*s > .31, η^2^ = .02), group by RT valence (all *p*s > .24, η^2^ = .03), or genotype by group by valence (all *p*s > .28, η^2^ = .03) These results suggest that the relation of depression group or genetic polymorphism on Eyes task accuracy reported below cannot be attributed to a speed-accuracy trade-off.

### Genes and Theory of Mind Decoding

#### SLC6A4 5-HTTLPR

The multivariate main effect of valence was significant, Wilks’ λ = .92, *F*(2, 91) = 3.89, *p* = .02, η^2^ = .08. This effect was qualified by significant interactions with depression group, Wilks’ λ = .89, *F* (2, 91) = 5.71, *p* = .005, η^2^ = .11, and genotype, Wilks’ λ = .91, *F* (2, 91) = 4.63, *p* = .01, η^2^ = .09. These were qualified by a significant 3-way interaction of valence, depression group, and genotype, Wilks’ λ = .90, *F*(2, 91) = 5.11, *p* = .008, η^2 =^ .10. Following up this 3-way interaction we determined that the valence by genotype interaction was not significant for the non-depressed group, Wilks’ λ = .97, *F*(2, 55) = .79, *p* = .46, η^2 =^ .03, but was significant for the depressed group, Wilks’ λ = .72, *F*(2, 35) = 8.98, *p =* .001, η^2 =^ .34. In the depressed group only, l/l homozygotes reported higher BDI-II scores than s-allele carriers at a trend (*M*s = 32.72, 28.01; *SD*s = 9.67, 5,55; *t*[35] = 1.88, *p* = .07). Thus, BDI-II scores were included as a covariate in the follow-up tests.

**Fig 2:**
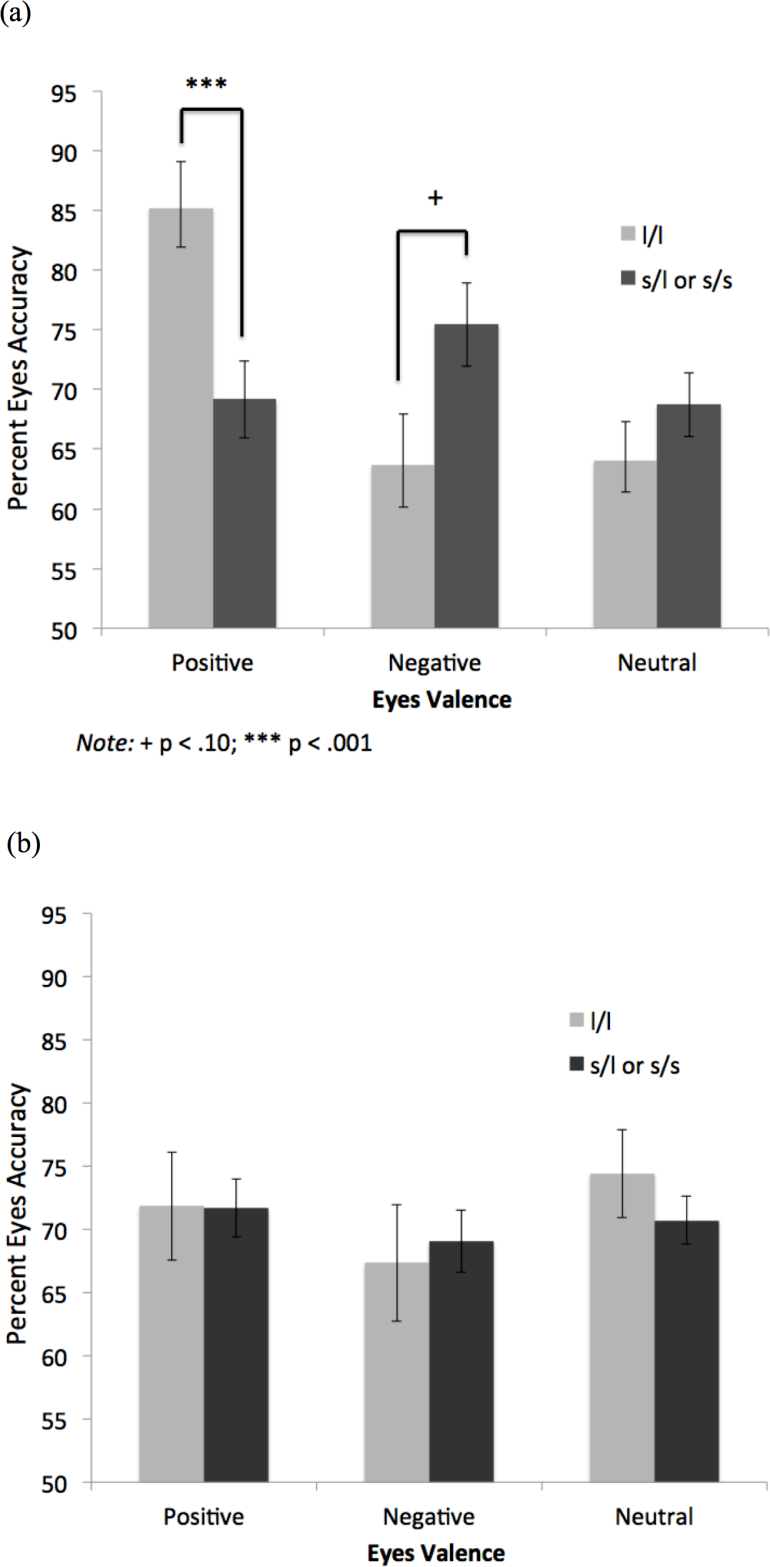
Eyes Accuracy by Valence and 5-HTTLPR Genotype in (a) Depressed and (b) Non-Depressed Groups.

**Fig 3:**
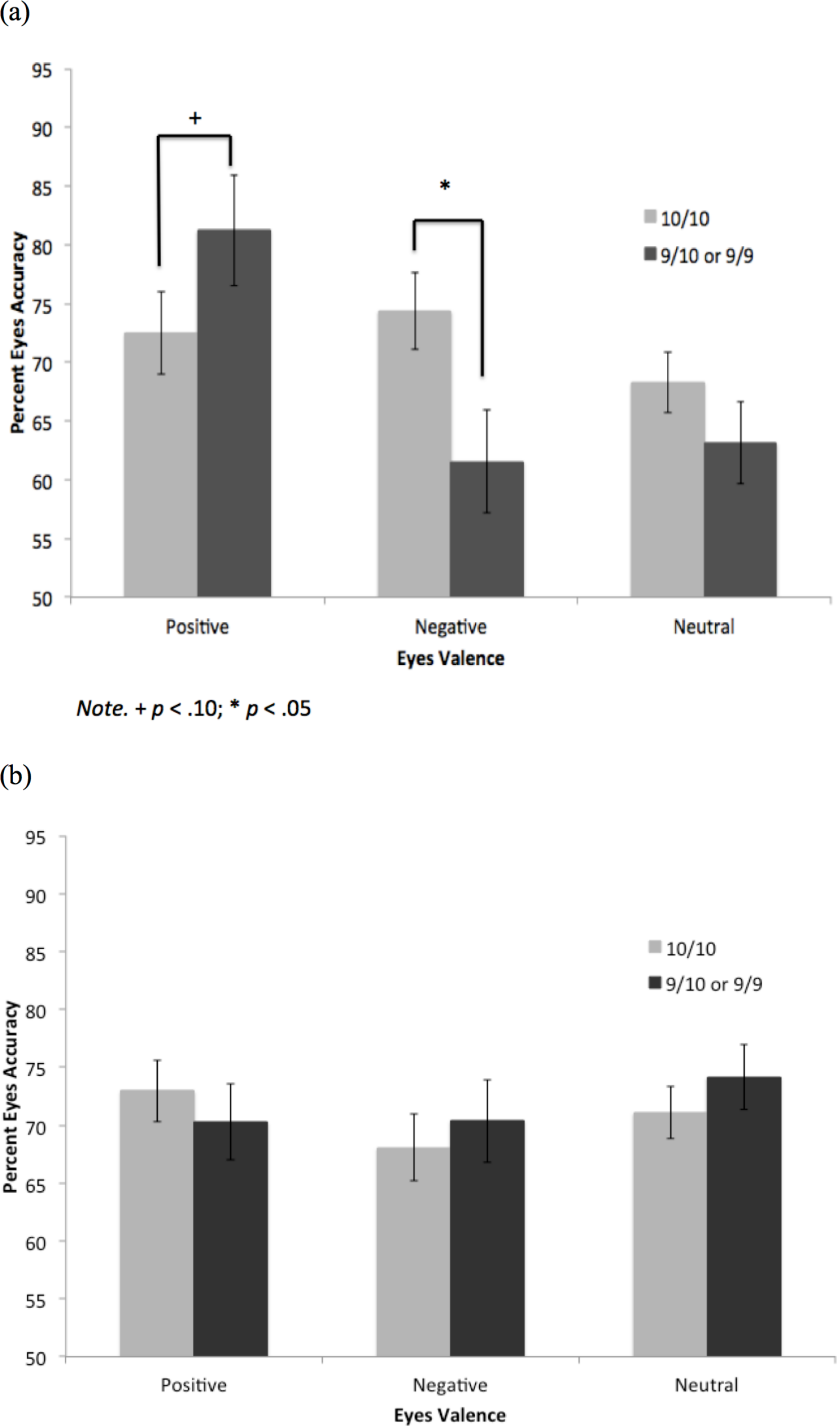
Eyes Accuracy by Valence and DAT1 VNTR Genotype in (a) Depressed and (b) Non-Depressed Groups.

**Fig 4:**
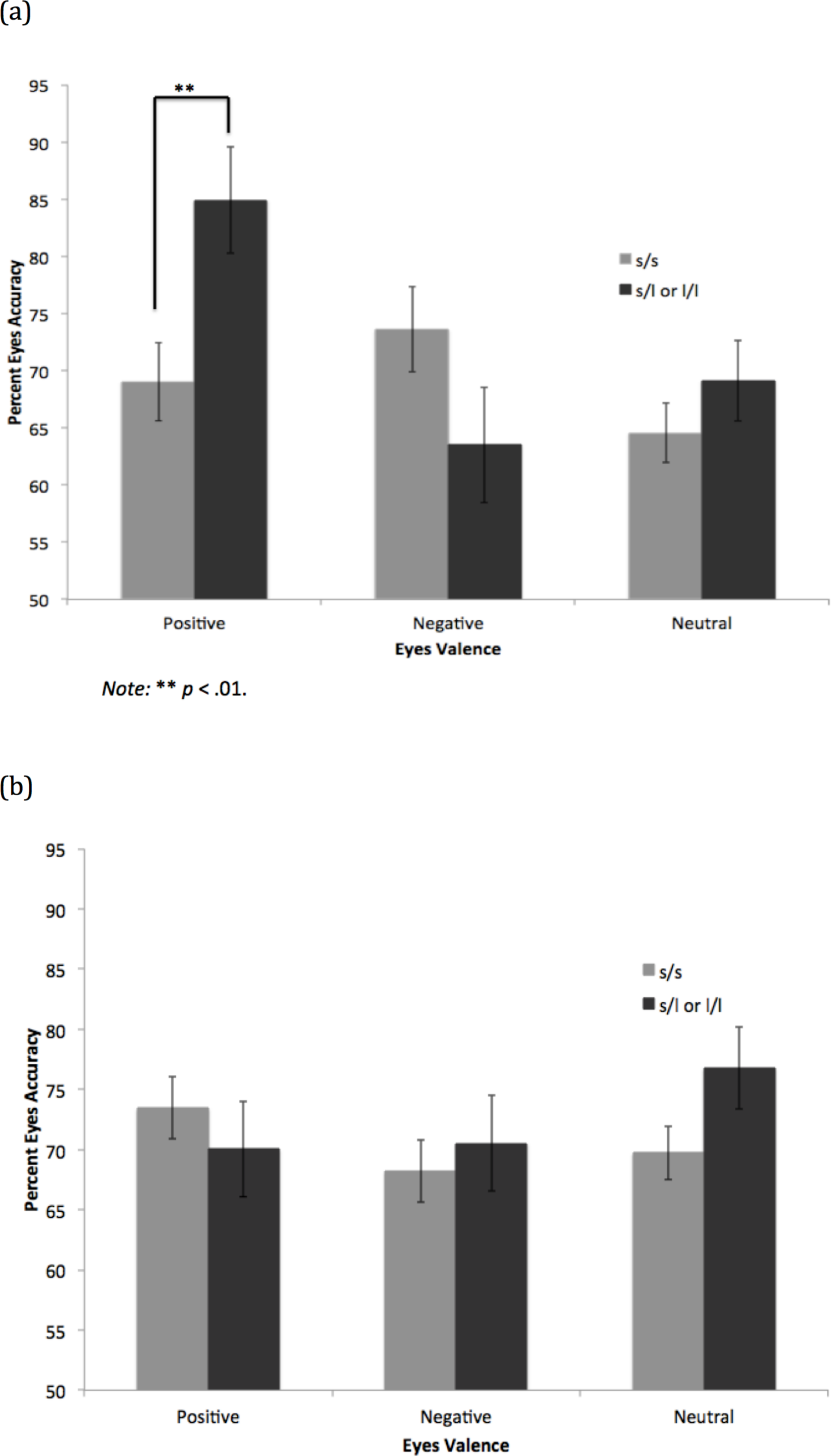
Eyes Accuracy by Valence and DRD4 VNTR Genotype in (a) Depressed and (b) Non-Depressed Groups.

In the MDD group, the l/l homozygotes were significantly more accurate than the s-allele carriers on the positive eyes, *F*(1, 34) = 19.78, *p* < .001, CI_95_ = 12.14, 32.57, η^2 =^ .37 (bootstrapped mean difference CI_95_ = 12.72, 30.16, *p* = .001) (Fig 2A; note that error bars in Figs 2A and 2B, 3A and 3B, and 4A and 4B represent standard errors of the mean). Further, there was a trend for the s-allele carriers to score higher than the l/l homozygotes on the negative eyes, *F*(1, 34) = 3.50, *p* = .07, CI_95_ = -.96, 23.08, η^2 =^ .09 (bootstrapped CI_95_ = -.08, 21.71, *p* = .06). The two groups did not differ significantly on the neutral eyes, *F*(1, 34) = .07, *p* = .80, η^2 =^ .002. Means of the non-depressed group are provided for comparison (Fig 2B).

#### DAT1 VNTR

Controlling for gender and ethnicity, the interaction of valence and depression group was significant, Wilks’ λ = .88, *F* (2, 85) = 5.57, *p* = .005, η^2^ = .12. This was qualified by a significant 3-way interaction of valence, depression group, and genotype, Wilks’ λ = .91, *F*(2, 85) = 4.06, *p* = .02, η^2 =^ .09. Following up this 3-way interaction we determined that the valence by genotype interaction was not significant for the non-depressed group, Wilks’ λ = .95, *F*(2, 51) = 1.39, *p* = .26, η^2 =^ .05, but was significant for the depressed group, Wilks’ λ = .78, *F*(2, 31) = 4.32, *p =* .02, η^2 =^ .22.

In the depressed group, the 10/l0 homozygotes were significantly more accurate than the 9-allele carriers on the negative eyes, *F*(1, 32) = 4.64, *p* = .04, CI_95_ = .72, 25.96, η^2 =^ .13 (Fig 3A) (bootstrapped CI_95_ = .03, 25.49, *p* = .048). While the 9-allele carriers scored than the 10/l0 homozygotes on the positive eyes, this difference was not significant, *F*(1, 32) = 3.32, *p* = .08, CI_95_ = -1.32, 23.80, η^2 =^ .06 (bootstrapped CI_95_ = -1.51, 23.66, *p* = .10). The two groups also did not differ significantly on the neutral eyes, *F*(1, 32) = .90, *p* = .35, η^2 =^ .03. Means of the non-depressed group are provided for comparison (Fig 3B).

#### DRD4 VNTR

Controlling for gender and ethnicity, the interaction of valence and depression group was significant, Wilks’ λ = .90, *F* (2, 86) = 4.98, *p* = .009, η^2^ = .10. Similar to above, in interaction with genotype, the valence by genotype interaction was not significant for the non-depressed group, Wilks’ λ = .92, *F*(2, 50) = 2.29, *p* = .11, η^2 =^ .08, but was significant for the depressed group, Wilks’ λ = .81, *F*(2, 33) = 3.91, *p =* .03, η^2 =^ .19.

In the depressed group, the long allele carriers were significantly more accurate than the short/short homozygotes on the positive eyes, *F*(1, 34) = 6.64, *p* = .01, CI_95_ = 3.35, 28.35, η^2 =^ .16 (see Fig 4A) (bootstrapped CI_95_ = 4.67, 25.93, *p* = .008). The two groups did not differ significantly on the negative eyes, *F*(1, 34) = 2.32, *p* = .14, η^2 =^ .06, or the neutral eyes, *F*(1, 34) = .97, *p* = .33, η^2 =^ .03. Means of the non-depressed group are provided for comparison (Fig 4B).

#### COMT Val158Met

Controlling for gender and ethnicity, the interaction of valence and depression group was significant, Wilks’ λ = .92, *F* (2, 89) = 3.59, *p* = .03, η^2^ = .08, such that the depressed group scored significantly lower than the non-depressed group on the neutral eyes, *M*s = 66.24, 71.96, *SD*s = 2.08, 1.69, *F*(1, 92) = 4.21, *p* = .04, η^2 =^ .04, but did not differ on either the positive or negative eyes (*p*s > .40, η^2^ < .007). However, there was no evidence for significant interactions of valence by COMT gene, Wilks’ λ > .99, *F*(2, 89) = .16, *p* = .85, η^2 =^ .004, or valence by COMT gene by depression, Wilks’ λ > .99, *F*(2, 89) = .09, *p* = .92, η^2 =^ .002.

## Discussion

In the current study we provide preliminary evidence that polymorphisms in serotonin and dopamine genes are associated with variation in ToM decoding in adults with MDD, whereas these same genetic variants are not significantly associated with ToM in healthy controls. Specifically, among participants with MDD only, superior accuracy in decoding mental states of a positive valence was seen in l/l homozygotes of the 5-HTTLPR, 9-allele carriers of DAT1VNTR (at a trend), and l-allele carriers of DRD4 VNTR. In contrast, superior accuracy in decoding mental states of a negative valence was seen in s-allele carriers of the SCL6A4 5-HTTLPR (at a trend) and 10/10 homozygotes of DAT1. These significant effects held over and above variance accounted for by sex, ethnicity, education level, and, importantly, response times and accuracy on the Animals control task, suggesting that our results are specific to the theory of mind decoding task and are not better accounted for by a speed-accuracy trade-off.

The association of COMT Val158Met to ToM decoding accuracy did not even approach significance, either as a main effect or in interaction with depression group. These null findings are consistent with those of previous studies [11, 17] and may suggest that processes of dopamine metabolism (associated with COMT) may not as centrally mediate ToM decoding skill as processes involving dopamine signaling in the synapse (associated with DAT1 and DRD4). In direct contrast, COMT and DRD4 were significantly associated as main effects with performance on the Animals control task. These effects are consistent with the very strong role of dopamine in executive function. In particular, studies in children, and in clinical syndromes such as schizophrenia, have consistently shown that those homozygous for the Val allele of COMT gene perform significantly more poorly on tasks of executive function than Met-allele carriers [13–14, 44–45]. Similarly, individuals homozygous for the long allele of the DRD4 gene perform significantly more poorly than short-allele carriers on measures of sustained attention, response inhibition, and other measures of executive function [46]. The Animals task includes an executive component. Specifically, respondents must keep competing and potentially relevant comparisons between the photo and the four adjectives in mind while determining which option is the correct response. Future studies are required to investigate further the extent to which differences in dopamine function and circuitry are implicated in processes of social cognition versus neurocognition more broadly construed.

The significant relations of genotype to ToM decoding emerged in the MDD group only. One possible reason for this is that MDD is associated with an altered baseline functioning of the neural circuitry that is associated with mental state decoding [22]. These baseline alterations may make depressed individuals especially susceptible to the effects of serotonergic and dopaminergic signaling. A similar phenomenon has been shown for dopaminergic functioning in children with phenylketonuria (PKU), for whom genetic effects are stronger than in typically developing children [47]. Similarly, as noted previously, the effect of serotonergic functioning on amygdala responses to valenced social stimuli is stronger in MDD patients than healthy controls [29]. Therefore, we may have had sufficient power to detect the large genetic association in the depressed group, but insufficient power to detect a weaker association in the healthy controls.

The effects for the 5-HTTLPR of the serotonin transporter gene were stronger than those for the dopamine genes, suggesting that serotonergic signaling may be more central to the decoding of mental states in MDD than is dopaminergic signaling. Given that the current study is the first to examine the relation of 5-HTTLPR polymorphisms to ToM, a consideration of the relation of these genetic variants to the more general skill of facial affect recognition may be helpful for interpretation of this effect. In healthy adult women, one previous study reported that 5-HTTLPR l/l homozygotes were significantly more accurate in the recognition of happy faces than s-allele carriers [48]. Further, 5-HTTLPR s-allele carriers were significantly more accurate than l/l homozygotes in the recognition of *fearful* faces, but not sad or angry faces. Similarly, in a recent imaging study of healthy women, greater neural activation in face processing regions was found for 5-HTTLPR l/l homozygotes in comparison to s-allele carriers in response to positive emotional stimuli [49]. In contrast, no differences in neural activation across polymorphisms were found for generally negative stimuli. These results, taken together with our findings, may suggest that the l/l genotype has effects on the processing of positive social stimuli generally (and for theory of mind, particularly in MDD). However, the effects of the s-allele on processing negatively valenced stimuli may be limited to socially *threatening* stimuli [19], thereby potentially accounting for the weaker relation of the 5-HTTLPR s-allele to the decoding of generally negative social stimuli in the current study. Future work examining the neurogenetic associations of ToM may benefit from more fine-grained categorizations of valence, particularly in the negative domain.

In terms of the dopaminergic genes, the DRD4 l-allele carriers in the MDD group demonstrated superior decoding of the positive eyes than MDD s/s homozygotes. The DRD4 l-allele has been associated with substance abuse and the rewarding effects of social context (e.g., social bonding) [50, 51], thus suggesting a role for this variant in the processing of rewarding stimuli. In contrast, DRD4 s/s homozygotes (at a trend) and DAT1 VNTR 10/10 homozygotes with MDD were more accurate at decoding mental states of a negative valence. These variants are associated with lower levels of dopamine signaling in the primary mesolimbic reward pathways.

The current results are partially consistent with previous work finding superior ToM *reasoning* skills in children who were homozygous for the s-allele of the DRD4 VNTR, but not for DAT1. DRD4 is strongly expressed in prefrontal regions [11]. This is consistent with Event-Related Potential (ERP) studies showing that theory of mind reasoning tasks activate dorsal-medial cortical regions over a long time-course [52]. In contrast, DAT1 is widely distributed in mesolimbic and mesocortical sites. ERP studies have associated ToM decoding tasks with a much quicker time course of neural activation over medial temporal sites [52, 53]. As such DAT1 may be more strongly involved in ToM decoding skill than ToM reasoning skill. This speculation is very preliminary, but underlines the point that ToM is not a monolithic process and instead is made up of multiple component parts operating at different levels of cognition [7]. This is an important point from a candidate gene association perspective. Breaking down complex behaviors into more targeted phenotypes with very specific neural circuitry and physiology, and then focusing on associations with genes that are most likely to have an affect on that neural circuitry and physiology maximizes the power. In other words, this approach is consistent with the strategy of identifying behaviors with as simple genetic architecture as possible for candidate gene studies. The results reported here suggest hypotheses that could drive future research aimed at clarifying the unique neurogenetic basis of different aspects of ToM skill.

The current results should be interpreted conservatively and in light of several limitations. First, our sample size was small for genetic studies and we report on four candidate gene associations, thereby raising the probability of reporting false positive results. Given the concerns over reliability of candidate gene studies on broader behavioral phenotypes, including those on more targeted phenotypes than binary diagnoses [54], we underscore the importance of presenting these results as preliminary findings. Consequently, direct replication of these associations in larger samples is required. Nevertheless, our choice of genes to target was theory-driven based on the highly specific neuroanatomical substrate of theory of mind. Second, our small sample size also prevented the consideration of gene by gene interactions in the above models, which may exist given the strong modulatory effect of serotonin on dopaminergic neurotransmission [10]. It is possible that the interaction of the polymorphisms under study and depression status on ToM reflects these epistatic relations. As a related limitation, the rs25531 SNP for the 5-HTTLPR has been shown to modify the effects of the long allele. In the current sample, however, examination of this SNP resulted in prohibitively low cell sizes for those homozygous for the long(a) allele. Therefore, an important question for future research with larger samples is whether the effects reported here replicate with the rs25531 SNP.

Third, the sample consisted of community volunteers, thus raising the possibility of spurious findings due to population substructure. Replication with a representative, epidemiological sample is warranted. Finally, this study focused on ToM decoding from eye expressions, thus future research is needed to determine if findings will generalize to other modalities (e.g., tone of voice). Future research is also needed to determine whether the current findings generalize to theory of mind reasoning about non-affectively valenced stimuli (e.g., reasoning about others’ beliefs or intentions). This will be important for determining whether the genetic associations reported here are specific to the modulation of affective information, *per se*, or extend to encompass non-affective social cognition.

In summary, the current study provides novel preliminary evidence that the heritability of ToM decoding might have a basis in genes that affect serotonergic and dopaminergic signaling, particularly in striatal or mesolimbic regions. Further, they suggest that such genetic effects on ToM decoding may be stronger in individuals with MDD relative to non-depressed individuals. The pattern of relation of serotonin and dopamine genes to enhanced sensitivity to others’ valenced mental states in MDD seen here is consistent with the relation of these genes to valenced biases in attention to, and recognition of, social stimuli, more broadly. These results, taken together, may ultimately have important implications for the role of social cognition in understanding the neuropathophysiology of MDD.

## Supporting Information

S1 Data Supporting Data set for Genes and Theory of Mind Analyses. (SAV)
